# Conceptual Invariance, Trajectories, and Outcome Associations of Working Alliance in Unguided and Guided Internet-Based Psychological Interventions: Secondary Analysis of a Randomized Controlled Trial

**DOI:** 10.2196/35496

**Published:** 2022-06-21

**Authors:** Xiaochen Luo, Matteo Bugatti, Lucero Molina, Jacqueline L Tilley, Brittain Mahaffey, Adam Gonzalez

**Affiliations:** 1 Department of Counseling Psychology Santa Clara University Santa Clara, CA United States; 2 Morgridge College of Education, University of Denver Denver, CO United States; 3 Department of Psychiatry, Stony Brook University Stony Brook, NY United States; 4 Psychological and Child & Human Development Area Group National Institute of Education Nanyang Technological University Singapore Singapore

**Keywords:** working alliance, internet-based psychological interventions, video support, text support, trajectory, MyCompass

## Abstract

**Background:**

The role of working alliance remains unclear for many forms of internet-based interventions (IBIs), a set of effective psychotherapy alternatives that do not require synchronous interactions between patients and therapists.

**Objective:**

This study examined the conceptual invariance, trajectories, and outcome associations of working alliance across an unguided IBI and guided IBIs that incorporated clinician support through asynchronous text messaging or video messaging.

**Methods:**

Adults with high education attainment (n=145) with subclinical levels of anxiety, stress, or depressive symptoms were randomized to 1 of 3 treatment conditions for 7 weeks. All participants received treatments from MyCompass, an unguided IBI using cognitive behavior therapy. Participants in condition 2 and 3 received supplemental, asynchronous clinician support through text and video, respectively. Working alliance with the IBIs was measured weekly using select items from the 12-item version of the Agnew Relationship Measure. Symptom and functional outcomes were assessed at baseline, at the end of treatment, and 1-month follow-up.

**Results:**

Working alliance with the IBIs was conceptually invariant across the 3 conditions. Working alliance followed a quadratic pattern of change over time for all conditions and declined significantly only in the text-support condition. After controlling for baseline symptoms, higher baseline levels of working alliance predicted less depression and less functional impairment at follow-up, whereas faster increases in working alliance predicted less worry at the end of treatment and at follow-up, all of which only occurred in the video-support condition.

**Conclusions:**

Working alliance with the IBIs was generally established in the initial sessions. Although working alliance is conceptually invariant across IBIs with or without clinician support, the associations between working alliance and treatment outcomes among IBIs may differ depending on clinician involvement and the modalities of support.

**Trial Registration:**

ClinicalTrials.gov NCT05122429; https://clinicaltrials.gov/ct2/show/NCT05122429

## Introduction

### Background

Working alliance is often conceptualized as a tripartite construct comprising agreement on therapeutic tasks and goals, as well as the bond between patients and therapists [1]. It has been identified as one of the most robust factors contributing to therapeutic change, with higher working alliance often associated with better treatment outcomes [2]. These effects have been consistently present in in-person psychotherapy as well as synchronous teletherapy, both of which feature direct, face-to-face interactions between clinicians and patients in real time [3]. However, the accessibility of in-person psychotherapy or synchronous teletherapy is limited by the shortage of clinicians, scheduling issues, difficulties with finding therapeutic space for both patients and therapists, transportation challenges (for in-person therapy), instability of internet connection (for synchronous teletherapy), perceived stigma of psychotherapy, and the financial cost of treatment. These barriers to in-person or synchronous teletherapy are especially salient during times of public health crisis, such as the COVID-19 pandemic, when the need for mental health services has surged despite limited supply.

Technology innovations have offered alternative options such as internet-based interventions (IBIs), which provide accessible mental health services that do not require synchronous communication. An unguided IBI, which is characterized by the delivery of a web-based therapeutic program with no support from clinicians, is an example of such interventions. Many unguided IBIs, also known as self-help programs, are based on cognitive behavior therapy (CBT) principles and involve components of psychoeducation, behavioral and cognitive practice, homework, and tracking-related activities for important variables (eg, mood and behaviors) [4,5]. In contrast to the absence of clinician involvement in unguided programs, guided IBIs often include a self-help program and asynchronous support from clinicians. Such integrated interventions are thought to improve treatment outcomes by leveraging the benefits of the therapeutic relationship between patients and clinicians. Clinicians in guided IBIs often provide low-intensity clinical guidance to facilitate the patient’s independent work with the self-help program [6]. Empirical studies have shown effectiveness for both types of IBIs in treating issues such as anxiety, depression, and traumatic stress for a wide range of populations [3,7,8], supporting their flexible use in situations where in-person communication or real-time telecommunication may be limited. However, what remains unclear is the nature and function of working alliance in guided and unguided IBIs.

### Conceptualization of Working Alliance and the Measurement Invariance Across IBIs

Previous studies have focused on understanding the level, trajectories, and outcome associations of working alliance in IBIs. A high level of working alliance has been reported by patients across guided IBIs with varying degrees of clinician involvement and different communication modalities [3,9], as well as across unguided programs [10]. Nonetheless, very few studies have directly compared the levels of working alliance between guided and unguided IBIs [11]. Such direct comparisons are needed to clarify (1) the nature of the relationships that patients have established with unguided intervention programs and (2) whether guided IBIs are able to improve outcomes by leveraging the benefits of working alliance with additional clinician support compared with unguided programs [12]. Information regarding these questions will help to determine the contexts in which additional clinician support is needed to improve the outcomes and delivery of IBIs.

However, a conceptual question arises regarding whether we can quantitatively compare working alliance between guided interventions and unguided interventions. Most previous studies have directly taken measures of working alliance from studies of face-to-face therapy with minor adaptations to IBI contexts (eg, replacing the word *therapist* with the word *program* in items to refer to the relationship with therapeutic programs and clinicians together). The potential differences of working alliance in various IBI contexts remained unexamined in most cases (although there are exceptions [13-15]). The conceptual meaning and interpretation of working alliance for patients may be different for unguided interventions versus guided interventions; unless we verify that working alliance has the same conceptual meaning across different IBI contexts, quantitative comparisons of working alliance across IBIs are meaningless. The measurement invariance framework using multigroup confirmatory factor analysis [16] provides a strong methodological tool to examine the conceptual equivalence of working alliance across contexts. Therefore, the first aim of the study was to examine the measurement invariance of working alliance across guided versus unguided IBIs.

In addition to the potential differences in working alliance between guided and unguided interventions, the nature of working alliance may also vary for guided IBIs with different communication modalities. Clinician support can be delivered through video-based messages or text messages, both of which may have unique impacts on the development of working alliance. For example, video-based support is hypothesized to better facilitate the development of working alliance than text-based support because it allows for visual messages with facial expressions that facilitate nonverbal communication and relational bonding [3]. By contrast, text-based support may allow participants to develop more thorough, in-depth responses through words, which can facilitate the establishment of working alliance by enhancing deep emotional processing [3]. Understanding the impact of different communication modalities can help to improve the design of IBIs and maximize the influence of clinician support on the therapeutic process. Therefore, in this study, we aimed to examine the measurement invariance among (1) an unguided IBI (U-IBI), (2) a guided IBI with text-based clinician support (G-IBI-Text), and (3) a guided IBI with video-based clinician support (G-IBI-Video).

### Trajectories of Working Alliance in IBIs

The literature on working alliance in face-to-face psychotherapy has consistently suggested that not only the levels but also the trajectories of working alliance matter. For example, studies show that varying trajectories of rupture repair–related patterns of working alliance in face-to-face therapy are differentially related to therapy progress [17], suggesting that the trajectories of working alliance are important for treatment outcomes. However, little is known regarding the trajectories of working alliance in IBIs. The study by Jasper et al [18] examined working alliance in guided IBIs and found that working alliance seemed to be low in the initial weeks of treatment and gradually increased during and at the end of treatment. This suggests that working alliance may generally increase over time for guided IBIs [18]. More studies are needed to examine the trajectories of working alliance developed in both guided and unguided IBIs to understand the development of working alliance and its potential impact on treatment outcomes. Therefore, this study’s second aim was to examine the trajectories of working alliance over the course of treatment in both guided and unguided IBIs.

### Associations Between Working Alliance and Treatment Outcomes in IBIs

Many studies have examined the associations between working alliance and treatment outcomes, but the field has not yet reached a consensus regarding this relationship in the context of IBIs. Several systematic reviews suggest mixed relationships between working alliance and treatment outcomes assessed at the end of treatment [3,9,11] and emphasize that the heterogeneity in clinician involvement and support modalities may contribute to the inconsistent results. Nonetheless, a recent meta-analysis [19] that summarized associations between working alliance and treatment outcomes in IBIs across 20 studies found an average weighted effect size of *r*=0.20 (95% CI 0.14-0.26) for the associations. It also noted that there was no difference between clinician communication modalities (ie, written formats such as email or text compared with oral formats such as telephone or video) or between interventions with or without self-help components (ie, interventions with no self-help components versus interventions that incorporated clinician support and self-help programs). However, no comparisons of working alliance and treatment outcome associations between unguided and guided programs were included in the study. Furthermore, the meta-analysis found significant higher associations between working alliance and treatment outcomes when working alliance was measured at the end of treatment rather than during the early phase of treatment, which indicated that the working alliance trajectories may influence the associations between working alliance and treatment outcomes. In light of these results, our third study aim was to examine the associations between treatment outcomes and trajectories of working alliance in both unguided and guided IBIs.

### Summary and Aims of This Study

In summary, there were 3 key aims of this study. First, we examined the measurement invariance of working alliance across 3 conditions of CBT-based IBIs (U-IBI, G-IBI-Text, and G-IBI-Video). We hypothesized that working alliance would be conceptually equivalent across the 3 conditions. Second, we examined the trajectories of working alliance over the course of the brief treatments in the 3 conditions. We expected to see increases in working alliance over time for all conditions. Finally, we examined the associations between working alliance trajectories and treatment outcomes (eg, mental health symptoms and functional impairment) across the 3 conditions. We hypothesized that higher working alliance would predict better treatment outcomes in all 3 conditions.

## Methods

### Study Design

We conducted secondary data analysis of a randomized controlled trial of a 7-week internet-based psychological intervention. The original study was a 3-arm randomized controlled trial that was designed to approximate treatment situations for treatment-seeking adults in stressful occupations and with no resources for real-time communications (eg, astronauts). Condition 1 was the U-IBI condition in which participants used an unguided, self-help IBI called *MyCompass* without additional clinician support. Condition 2 was the G-IBI-Text condition in which participants used the same self-help IBI (*MyCompass*) and received additional asynchronous text-based support from a clinician. Condition 3 was the G-IBI-Video condition in which participants used the same self-help IBI (*MyCompass*) and received additional asynchronous video-based support from a clinician.

### Ethics Approval

The study design and protocol were approved by the Stony Brook University Institutional Review Board (903034).

### Interventions and Clinicians

The *MyCompass* program is a self-help IBI designed and shown to improve mild to moderate symptoms of depression, anxiety, and stress [20]. The program offers 14 self-management modules based on CBT principles, each of which comprises 3 sessions lasting 10 minutes each. The *MyCompass* program also includes homework tasks for each module as well as functions such as mood and symptom tracking, feedback from the program on patient performance, and psychoeducation (refer to Figures S1-S4 in Multimedia Appendix 1 for the interface and examples of *MyCompass*).

Participants in all 3 conditions were asked to complete at least two modules of their choice on *MyCompass* during the 7-week treatment period. Participants in the U-IBI condition received automated email reminders to encourage them to use the program, track their symptoms, examine patterns and triggers related to changes in their mood and behaviors, and practice the skills they learned in real-world situations. No clinician support was provided in this condition.

Each participant in the G-IBI-Text condition was assigned to a clinician for additional, asynchronous text-based support. All clinicians (n=10) had master’s or higher-level degrees and were trained and supervised weekly by 2 licensed psychologists (BM and AG). The clinicians initiated 1 weekly message through text at a prescheduled time to provide general support and positive reinforcement for program participation. This text-based contact typically involved encouraging participants to log on to *MyCompass* or to try a *MyCompass* module that was relevant to a stressor identified by the participant in a previous message to the clinician. Clinicians were instructed not to introduce skills or concepts not covered by the *MyCompass* program. Participants could respond to their clinician or initiate contact with them at any time during the 7-week study but were informed that clinicians would only respond to messages during specified business hours. Clinicians were encouraged to use their own words rather than prefabricated responses to communicate with their patients.

The experience of participants in the G-IBI-Video condition was similar to that of participants in the G-IBI-Text condition, except that the clinicians initiated a weekly *video* message and communicated with participants through asynchronous video messages. Participants could initiate contact with clinicians or respond to clinicians by sending video messages on a communication platform that was specifically designed to receive asynchronous video messages for this study. As in the G-IBI-Text condition, clinicians in the G-IBI-Video condition were instructed not to introduce skills or concepts not covered by the *MyCompass* program.

### Participants

Adults with high education attainment who sought treatment for subclinical levels of anxiety, depression, and stress were selected in the original study. The inclusion criteria were as follows: (1) aged ≥18 years; (2) English speaking; (3) enrolled in, or completed, a graduate-level education in science, technology, engineering, or math domains; (4) having a score of ≥5 on the depression subscale, ≥4 on the anxiety subscale, or ≥8 on the stress subscale of the 21-item version of the Depression, Anxiety, and Stress Scale [21], which indicates a moderate or higher level of clinical symptoms; and (5) having a score of ≥5 on any subscales or ≥6 on the global scale of the Sheehan Disability Scale (SDS), which indicates a moderate or higher level of functional impairment [22].

The exclusion criteria were as follows: (1) active suicidal ideation in the past month, (2) any history of suicide attempt within the past 5 years, (3) having a diagnosis of psychotic disorder or bipolar disorder, (4) alcohol or substance dependency in the past 6 months, (5) serious medical problems (eg, seizures or cancer), (6) pregnancy, (7) current participation in psychotherapy, and (8) having recently started a new psychoactive medication (ie, benzodiazepines for <1 month or selective serotonin reuptake inhibitors, tricyclics, or serotonin-norepinephrine reuptake inhibitors for <3 months). The Mini International Neuropsychiatric Interview [23] was used for assessing study eligibility.

Eligible participants completed a baseline assessment consisting of in-person or over-the-telephone clinical assessment and web-based questionnaires administered through Qualtrics. After this baseline assessment, participants were randomly assigned to a condition based on a pregenerated random assignment table—the procedure was weighted to favor assignment to the U-IBI condition. Participants in all 3 conditions received access to the *MyCompass* program and the study web-based portal.

A total of 300 individuals completed screening forms to indicate interest in the study between May 2018 and September 2018. Of the 300 individuals screened, 155 (51.7%) were excluded for the following reasons: they did not meet the inclusion criteria (n=146, 94.2%), were no longer interested when contacted by the research team (n=4, 2.6%), or met an exclusion criterion (n=5, 3.2%). Subsequently, of the 300 people screened, 145 (48.3%) were enrolled into the trial. These 145 participants were randomized to the U-IBI condition (n=57, 39.3%), the G-IBI-Text condition (n=44, 30.3%), or the G-IBI-Video condition (n=44, 30.3%; refer to Multimedia Appendix 2 for the CONSORT [Consolidated Standards of Reporting Trials] flow diagram).

Most of the 145 participants were women (n=99, 68.3%), of heterosexual orientation (n=125, 86.2%), and identified as White (n=99, 68.3%) and non-Hispanic (n=133, 91.7%). The average age was 30 (SD 8.21) years. In total, 96.6% (140/145) of the participants were college graduates, with 59.3% (86/145) having at least a master’s degree. The proportion of participants identifying as Hispanic was lower in the G-IBI-Text condition than in the other 2 treatment conditions; otherwise, no demographic differences in sex, age, sexual orientation, race, ethnicity, or education attainment were noted across the 3 conditions (refer to Multimedia Appendix 3 for the demographic characteristics of the participants).

### Measures

#### Working Alliance

In total, 4 items that were adapted from the Agnew Relationship Measure, 12-item version (ARM-12) were used to examine working alliance across the treatment conditions [24]. The ARM-12 has been widely used to examine working alliance in face-to-face therapy and has shown a strong reliability and good criterion validity with other working alliance measures [25,26]. The ARM-12 is one of the most commonly used questionnaires to assess working alliance in internet-based mental health interventions because of its conciseness and its full representation of the relevant concepts [27-30]. However, most studies have adapted the ARM-12 for IBIs by simply changing the term *clinician* to *program* or *app* in items [13]. Such alteration of wording may create issues with content validity (ie, an original item such as “the *clinician* seems bored or impatient with me” is modified to “the *program* seems bored or impatient with me”). Therefore, to enhance the measure’s content validity across the treatment conditions, this study only included items that were assessed by experts’ and users’ consensus in previous qualitative studies as relevant for IBIs [13]. The 4 included items were as follows: “I feel friendly toward the program,” “I have confidence in the program and its techniques,” “I feel I can openly express my thoughts and feelings to the program,” and “The program is supportive.” Each item was rated using a 7-point Likert scale (from 1=*strongly disagree* to 7=*strongly agree*). The ARM-12 items were administered after each week of the intervention, starting from the first week and ending in the seventh week.

The Cronbach α values were .78 and .91 for the 4 items assessed at week 1 and at the end of treatment, respectively. We also examined factorial validity in confirmatory factor analysis for a single common factor of these 4 items at baseline and at the end of treatment, given the previous finding of a core working alliance factor for short versions of the ARM [24]. The single common factor model fit perfectly for the 4 items assessed at week 1 and at the end of treatment (*χ*^2^_2_=1.2 and *χ*^2^_2_=1.3, respectively; comparative fit index=1.00, Tucker-Lewis Index=1.00, and root mean squared error of approximation (RMSEA)=0.00 in models at week 1 and at the end of treatment), suggesting that a single common factor of the working alliance underlay the 4 items.

#### Treatment Outcomes

The Patient Health Questionnaire-9 (PHQ-9) [31], Penn State Worry Questionnaire (PSWQ) [32], and SDS [22] were used as treatment outcome measures to assess depression, anxiety, and social functioning impairment, respectively, at baseline, at the end of treatment, and 1-month follow-up. The PHQ-9 is a self-report measure for general depression symptoms, with higher scores indicating more depressive symptoms. The Cronbach α values for the internal consistency were .79, .87, and .88 in our sample at baseline, at the end of treatment, and 1-month posttreatment follow-up, respectively. The PSWQ is a 21-item measure for worry symptoms, with higher scores indicating higher levels of worry. The Cronbach α values for the internal consistency were .76, .82, and .79 in our sample at baseline, at the end of treatment, and 1-month posttreatment follow-up, respectively. The SDS is a 3-item measure for social functioning impairment. The Cronbach α values for the internal consistency were .73, .89, and .89 in our sample at baseline, at the end of treatment, and 1-month posttreatment follow-up, respectively.

### Data-Analytic Strategy

#### Overview

Multigroup confirmatory factor analysis and multigroup longitudinal structural equation modeling were used to assess the conceptual invariance, trajectories, and treatment outcome associations across treatment conditions. Data were modeled with Mplus (version 8.2; Muthén & Muthén) [33]. Full information maximum likelihood estimation [34] was used to handle missing data. We evaluated and compared the model fit for all models based on 6 model indices: chi-square [35], comparative fit index [36] (values >0.90 indicate acceptable fit), Tucker-Lewis Index [36] (values >0.90 indicate acceptable fit), RMSEA [37] (values <0.08 indicate acceptable fit), Akaike information criterion (lower values indicate better fit), and Bayesian information criterion (lower values indicate better fit). We compared nested models by calculating a chi-square difference test such that a nonsignificant chi-square difference indicates a preference for the nested, more parsimonious model.

#### Conceptual Invariance of Working Alliance

Confirmatory factor analysis was used to evaluate measurement invariance and determine whether working alliance was conceptually comparable across the 3 IBI conditions. In the multigroup invariance analyses, baseline models with no constraints requiring equality among the groups were compared with various invariance models to determine the best modeling fit to the data [38]. We used a single-factor baseline model with no restraints, requiring equality among the groups as the baseline model, and compared this model with three types of alternative, invariance models: (1) a configural invariance model (testing a single-factor model in all treatment groups without constraining the factor loadings or intercepts), which indicates the same conceptual factor structure across treatment groups; (2) a metric invariance model (constraining the factor loadings to be equivalent across groups), which indicates that in addition to the same factor structure of the single-factor model, these groups have the same factor loadings on the single factor; and (3) a scalar invariance model (constraining both factor loadings and intercepts to be equivalent), which indicates that the 3 conditions have the same factor structure as the single-factor model, the same factor loadings, and the same true values on the latent factor. If the scalar invariance model is the best-fitting model, it indicates that the working alliance is conceptually invariant and that the latent values can be compared across treatment conditions [39]. We examined measurement invariance separately for working alliance assessed at the first week of treatment and at the end of treatment.

#### Trajectories of Working Alliance

Once we determined that working alliance was conceptually invariant (refer to the *Results* section), we fit a series of univariate latent growth curve models to identify the appropriate change pattern of working alliance for the entire sample. Data were modeled with multiple types of trajectories, including (1) a no-change, intercept-only model where we only estimated means and variance for all measurement points without a slope; this model indicates no change in working alliance over time; (2) a linear change model where we estimated both intercept and slope for the trajectories; this model indicates that working alliance changes in a linear fashion over time; (3) a latent basis model, where an intercept and a slope were estimated but the loading on the slope was not based on the temporal time and is freely estimated; this model indicates that working alliance may change at a nonlinear rate with time; and (4) a quadratic model where we estimated the intercept, a linear slope, and a quadratic slope; this model indicates that working alliance may change in a quadratic pattern. We used the model fit indices to determine the best-fitting model that depicted the trajectories of working alliance among the aforementioned models.

After identifying the best-fitting model in which everything was set as equal across treatment conditions (ie, the fully constrained model), we created alternative models in which the 3 conditions may not be equal (by gradually loosening the constraints of the parameters) and compared the model fit between alternative models and the fully constrained model. The following parameters (if they existed in the best-fitting model) were loosened to be uniquely estimated in each group one at a time: mean of intercept, mean of linear slope, quadratic slope, mean of autoregressive coefficient (if it existed), variance of intercept, variance of linear slope, variance of quadratic slope, and residual variance. In case of model misspecifications, the cause of misspecification was examined through modification indices. Model modifications were used with caution and applied only if supported by possible theoretical explanations. If any of the alternative models yielded better model fit than the fully constrained model, it indicated differences in the trajectories of working alliance across treatment conditions.

#### Associations Between Working Alliance and Treatment Outcomes

We examined whether the trajectories of working alliance contributed to treatment outcomes in each condition by examining whether the intercept (the initial level) or the change rates (linear or quadratic slope, if identified in previous steps) of the working alliance would predict treatment outcomes on depression (PHQ-9), worry (PSWQ), and social functioning impairment (SDS). We did so by including each outcome and the associations between each outcome and the intercept and slope of working alliance in the best-fitting multigroup structural equation modeling models that were identified from the previous step. We ran separate models for each outcome and controlled for the baseline level of each treatment outcome measure in each model.

## Results

### Overview

**Figure 1:**
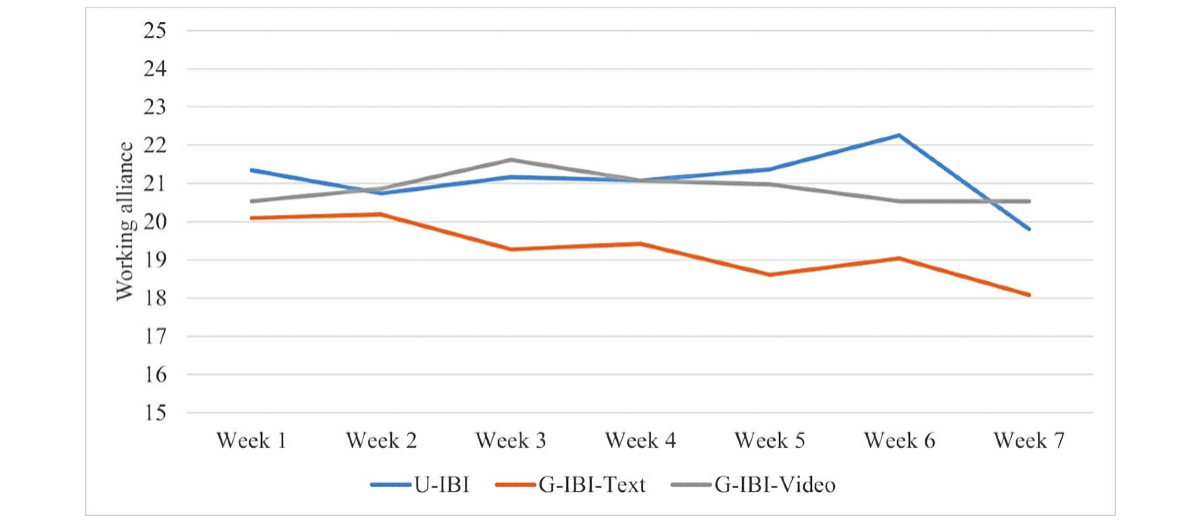
The weekly working alliance ratings across treatment for each treatment condition. U-IBI: unguided internet-based intervention; G-IBI-Text: guided internet-based intervention with text-based clinician support; G-IBI-Video: guided internet-based intervention with video-based clinician support.

The levels of working alliance based on the selected items of ARM-12 for week 1 to week 7 are presented in Figure 1. The treatment outcome variables were moderately correlated concurrently in the range of 0.48 to 0.66 between social functional impairment and depression and in the range of 0.43 to 0.50 between worry and depression and between worry and functional impairment.

### Conceptual Invariance of Working Alliance

The model fit indices for configural, metric, and scalar invariance models for the selected working alliance items at week 1 and week 7 are presented in Table 1. Overall, the scalar invariance model across the 3 treatment conditions reached an excellent model fit at both week 1 and week 7 (chi-square test for model fit; *P*=.56 and *P*=.05 in scalar invariance models for week 1 and for week 7, respectively). This suggests that the selected 4 items of the ARM-12 had measurement invariance across treatment groups, indicating that it is appropriate to compare scores across conditions to detect differences on the latent construct of working alliance.

**Table 1:** The model fit indices for configural, metric, and scalar invariance of working alliance (selected items from the Agnew Relationship Measure, 12-item version) across the 3 conditions at week 1 and week 7.

Assessment week and model			Free parameters, n		Chi-square (*df*)		*P* value		RMSEA^a^		CFI^b^		TLI^c^
**Week 1**													
	Configural invariance	25		18.9 (17)		.34		0.05		0.99		0.99	
	Metric invariance	19		22.0 (23)		.52		0.00		1.00		1.00	
	*Scalar invariance^d^*	*11*		*29.2 (31)*		*.56*		*0.00*		*1.00*		*1.00*	
**Week 7 (at the end of treatment)**													
	Configural invariance	25		29.5 (17)		.03		0.13		0.96		0.96	
	Metric invariance	19		35.8 (23)		.04		0.11		0.96		0.97	
	*Scalar invariance^d^*	*11*		*44.8 (31)*		*.05*		*0.10*		*0.96*		*0.98*	

^a^RMSEA: root mean squared error of approximation.

^b^CFI: comparative fit index.

^c^TLI: Tucker-Lewis Index.

^d^Text in italics indicates the best-fitting model selected.

### Trajectories of Working Alliance

The model fit indices for latent curve models are presented in Table 2. When we constrained the treatment groups to be fully equal (ie, assuming no group differences), the quadratic model was the best model for the entire sample with acceptable fit, except for a slightly elevated index with RMSEA.

This quadratic model for all groups was then used as the baseline model in the multigroup modeling comparison to examine whether there were any group differences in trajectories. We compared this model with alternative models where we allowed 1 parameter to be different at a time. We identified the best-fitting model based on the chi-square difference test, acceptable model fit indices (comparative fit index=0.95, Tucker-Lewis Index=0.97, RMSEA=0.09), and the lowest Akaike information criterion and Bayesian information criterion. The best-fitting model allowed the linear slope to be different in the G-IBI-Text condition but not in the other 2 conditions. In addition, the best-fitting model allowed the residual variance to be different in the G-IBI-Text condition. This indicated that for the best-fitting model, there was a significantly different linear change rate in the G-IBI-Text condition compared with the other 2 conditions.

The estimations for the parameters are presented in Table 3. Specifically, the G-IBI-Text condition had a significant linear slope that was negative (linear slope estimation=−0.44; *P*=.04) compared with the nonsignificant linear slope for the other 2 conditions (linear slope estimation=0.19; *P*=.35). This indicates that the working alliance followed a different quadratic pattern in the G-IBI-Text condition compared with the other 2 conditions, in that there was a significant linear decrease only in the G-IBI-Text condition and no significant linear change in working alliance for the U-IBI or G-IBI-Video conditions.

**Table 2:** Model fit indices for multigroup latent curve modeling of working alliance (selected items from the Agnew Relationship Measure, 12-item version).

Model		Free parameters, n	AIC^a^	BIC^b^	Chi-square (*df*)	RMSEA^c^	CFI^d^	TLI^e^	ΔChi-square^f^ (Δ*df*)	*P* value for Δchi-square (Δ*df*)
**Fully constrained models for the entire sample as 1 group**										
	Intercept only	3	3879.21	3888.07	183.5 (32)	0.18	0.79	0.86	N/A^g^	N/A
	Linear	6	3797.86	3815.59	96.1 (29)	0.13	0.91	0.93	87.4 (3)	<.001
	Latent basis	11	3793.24	3825.76	81.5 (24)	0.13	0.92	0.93	102.0 (8)	<.001
	*Quadratic^h^*	*10*	*3762.95*	*3792.51*	*53.2 (25)*	*0.09*	*0.96*	*0.97*	*130.3 (7)*	*<.001*
**Multigroup modeling allowing for group differences**										
	Baseline, fully constrained quadratic model	10	3762.95	3792.51	143.0 (95)	0.10	0.94	0.96	N/A	N/A
	Free intercept	12	3765.32	3800.79	141.3 (93)	0.11	0.94	0.96	1.4 (2)	.50
	Free linear slope	12	3762.08	3797.55	138.1 (93)	0.10	0.94	0.96	4.9 (2)	.09
	Free linear slope and quadratic slope	14	3765.39	3806.78	137.4 (91)	0.10	0.94	0.96	5.6 (4)	.23
	Free quadratic slope	12	3762.91	3798.37	138.9 (93)	0.10	0.94	0.96	4.0 (2)	.14
	Free variance and covariance	22	3777.70	3842.73	133.7 (83)	0.11	0.93	0.95	9.3 (12)	.68
	Free residual variance	12	3753.00	3788.62	129.2 (93)	0.09	0.95	0.97	13.8 (2)	.001
	Free residual variance and linear slope	14	3752.42	3793.80	124.0 (91)	0.09	0.96	0.97	19.0 (4)	<.001
	*Free residual and linear slope only for the text group^h^*	*12*	*3751.22*	*3786.69*	*127.2 (93)*	*0.09*	*0.95*	*0.97*	*15.7 (2)*	*<.001*
	Free linear slope only for the text group	11	3760.92	3793.43	138.9 (94)	0.10	0.94	0.96	4.0 (1)	.05
	Free residual variance only for the text group	11	3753.08	3785.59	131.1 (94)	0.09	0.95	0.97	11.9 (1)	<.001

^a^AIC: Akaike information criterion.

^b^BIC: Bayesian information criterion.

^c^RMSEA: root mean squared error of approximation.

^d^CFI: comparative fit index.

^e^TLI: Tucker-Lewis Index.

^f^ΔChi-square: chi-square difference test.

^g^N/A: not applicable (the chi-square difference test is not applicable to the baseline models).

^h^The models presented in italics indicated the best-fitting models in each category. We first fit models for the entire sample and identified the quadratic model as the best-fitting model. Next, we fit the quadratic model to the 3 conditions in multigroup structural equation modeling, constraining the parameters to be the same for each group. We then gradually loosened the constraints to examine alternative models. The best-fitting model for multigroup modeling indicated a model in which the residual variance and linear slope were set to be different for the guided internet-based intervention with text-based clinician support condition only.

**Table 3:** Parameter estimation in the best-fitting model.

Parameters	U-IBI^a^		G-IBI-Text^b^		G-IBI-Video^c^	
	Estimate (SE)	*P* value	Estimate (SE)	*P* value	Estimate (SE)	*P* value
Level factor means	20.65 (0.30)	<.001	20.65 (0.30)	<.001	20.65 (0.30)	<.001
Linear slope factor means	−0.19 (0.20)	.35	−*0.44^d^* (*0.22*)	*.04*	−0.19 (0.20)	.35
Quadratic slope factor means	0.01 (0.03)	.72	0.01 (0.03)	.72	0.01 (0.03)	.72
Level factor variance	9.42 (1.52)	<.001	9.42 (1.52)	<.001	9.42 (1.52)	<.001
Linear slope factor variance	2.68 (0.63)	<.001	2.68 (0.63)	<.001	2.68 (0.63)	<.001
Quadratic slope factor variance	0.06 (0.01)	<.001	0.06 (0.01)	<.001	0.06 (0.01)	<.001
Residual variance	3.05 (0.27)	<.001	*4.98 (0.61)*	*<.001*	3.05 (0.27)	<.001
Covariance between level factor and linear slope factor	0.74 (0.69)	.29	0.74 (0.69)	.29	0.74 (0.69)	.29
Covariance between level factor and quadratic slope factor	−0.11 (0.10)	.28	−0.11 (0.10)	.28	−0.11 (0.10)	.28
Covariance between linear slope factor and quadratic slope factor	−0.37 (0.09)	<.001	−0.37 (0.09)	<.001	−0.37 (0.09)	<.001

^a^U-IBI: unguided internet-based intervention.

^b^G-IBI-Text: guided internet-based intervention with text-based clinician support.

^c^G-IBI-Video: guided internet-based intervention with video-based clinician support.

^d^The parameters in the 3 conditions were fixed to be the same, except for the ones in italics, which were estimated separately for the guided internet-based intervention with text-based clinician support condition.

### Associations Between Working Alliance and Treatment Outcomes

We examined how the intercept (ie, initial level) and the linear slope (ie, the linear change rate) of the working alliance trajectories predicted each treatment outcome (depression, worry, and functional impairment) at the end of treatment and at 1-month follow-up after controlling for each treatment outcome variable at baseline separately. The model fit indices are presented in Table 4. All models reached acceptable fit. The parameter estimations are shown in Table 5.

**Table 4:** Model fit indices for multigroup models with outcomes^a^ at the end of treatment and 1-month follow-up.

Model	Outcome	Assessment	Free parameters, n	Chi-square (*df*)	RMSEA^b^	CFI^c^	TLI^d^
Model 1	PHQ-9^e^	the end of treatment	27	168.4 (129)	0.08	0.95	0.96
Model 2	PHQ-9	1-month follow-up	27	176.4 (129)	0.09	0.94	0.95
Model 3	PSWQ^f^	the end of treatment	27	172.6 (129)	0.09	0.95	0.96
Model 4	PSWQ	1-month follow-up	27	171.1 (129)	0.08	0.95	0.96
Model 5	SDS^g^	the end of treatment	27	182.4 (129)	0.09	0.93	0.94
Model 6	SDS	1-month follow-up	27	163.8 (129)	0.08	0.95	0.96

^a^The parameters indicated a good fit for all the models incorporating outcome measures.

^b^RMSEA: root mean squared error of approximation.

^c^CFI: comparative fit index.

^d^TLI: Tucker-Lewis Index.

^e^PHQ-9: Patient Health Questionnaire-9.

^f^PSWQ: Penn State Worry Questionnaire.

^g^SDS: Sheehan Disability Scale.

**Table 5:** The parameter estimations for alliance-outcome associations.

SEM^a^ model and predictors							Outcomes		U-IBI^b^								G-IBI-Text^c^						G-IBI-Video^d^			
									Estimate				*P* value				Estimate			*P* value		Estimate				*P* value
**PHQ-9^e^**																										
	**Model 1**																									
			Intercept of alliance				PHQ-9 at the end of treatment		−0.06						.77		−0.1			.71		−0.02				.91
			Linear slope of alliance				PHQ-9 at the end of treatment		−0.44						.35		0.28			.65		−0.8				.09
	**Model 2**																									
			Intercept of alliance		PHQ-9 at 1-month follow-up						0.05	.86				0.004			.99		−*0.62^f^*				*.04*	
			Linear slope of alliance		PHQ-9 at 1-month follow-up						−0.82	.14				0.43			.42		0.04				.95	
**PSWQ^g^**																										
	**Model 3**																									
		Intercept of alliance		PSWQ at the end of treatment						0.59				.14				0.01	.99		0.83			.18		
		Linear slope of alliance		PSWQ at the end of treatment						−*1.8*				*.05*				0.82	.46		−*2.85*			*.03*		
	**Model 4**																									
		Intercept of alliance		PSWQ at 1-month follow-up						0.18				.62				−0.95	.08		0.67			.29		
		Linear slope of alliance		PSWQ at 1-month follow-up						−0.67				.40				2.17	.08		−*3.61*			*.01*		
**SDS^h^**																										
	**Model 5**																									
		Intercept of alliance				SDS at the end of treatment		−0.42				.16				−0.47			.14		−0.55				.17	
		Linear slope of alliance				SDS at the end of treatment		0.59				.36				0.48			.51		−0.34				.72	
	**Model 6**																									
		Intercept of alliance				SDS at 1-month follow-up		0.07				.82				−0.62			.14		−*0.76*				*.04*	
		Linear slope of alliance				SDS at 1-month follow-up		−0.004				.99				0.85			.39		0.64				.43	

^a^SEM: structural equation modeling.

^b^U-IBI: unguided internet-based intervention.

^c^G-IBI-Text: guided internet-based intervention with text-based clinician support.

^d^G-IBI-Video: guided internet-based intervention with video-based clinician support.

^e^PHQ-9: Patient Health Questionnaire-9.

^f^Italicized items indicate significance at *P*<.05 level.

^g^PSWQ: Penn State Worry Questionnaire.

^h^SDS: Sheehan Disability Scale.

After controlling for baseline severity, the associations between treatment outcomes and working alliance seemed to vary across conditions. In the G-IBI-Video condition, the intercept of working alliance significantly predicted depression (PHQ-9) and functioning impairment (SDS) negatively at 1-month follow-up (*P*=.04 for both models) such that a higher initial level of working alliance predicted lower depression and lower functional impairment for the participants at 1-month follow-up after controlling for their baseline levels of depression or functional impairment. In addition, for the G-IBI-Video condition, the linear slope of working alliance negatively predicted worry (PSWQ) at the end of treatment and at 1-month follow-up (*P*=.03 and *P*=.009, respectively), which means that a faster increase of working alliance over time would predict less worry at the end of treatment and at 1-month follow-up, after controlling for baseline levels of worry.

By contrast, neither the intercept nor the linear slope of working alliance was associated with any treatment outcomes at either time points for the U-IBI condition or the G-IBI-Text condition, with 1 exception: the linear slope of working alliance was negatively associated with worry (PSWQ) at the end of the treatment (*P*=.049) in the U-IBI condition. However, this significant effect disappeared at 1-month follow-up.

## Discussion

### Summary

Internet-based psychological interventions promise to overcome accessibility-related issues associated with face-to-face, synchronous interventions while also embracing a patient-centered and stepped-care approach to mental health services. Despite a growing body of research supporting the efficacy and effectiveness of IBIs, little is known regarding the relevance of psychotherapy relationship factors, such as working alliance, which are known to account for a significant portion of in-person treatment outcomes. Moreover, although some studies have examined the effects of the inclusion of clinician support services in IBIs, to our knowledge, no existing investigation has directly compared the effects of varying degrees of clinician involvement in IBIs on working alliance. This study was designed to address this knowledge gap by examining the measurement invariance and trajectories of working alliance, as well as the associations between working alliance and treatment outcomes across an unguided IBI and guided IBIs with text-based or video-based clinician support. We found that although the conceptual interpretation and the trajectories of working alliance was relatively similar across the 3 conditions, higher working alliance predicted better treatment outcomes only in the video-support (G-IBI-Video) condition.

### Conceptual Invariance of Working Alliance

The results indicated that participants’ ratings of their working alliance with the IBIs were conceptually invariant across the U-IBI condition and the asynchronous G-IBI-Text and G-IBI-Video conditions. This result was consistent with our hypothesis, supporting the equivalence of the underlying construct of working alliance for IBIs and allowed for further between-group comparisons across conditions. A growing number of studies [14,40] have explored the applicability of the tripartite construct of alliance formulated by Bordin [1] to IBIs. This study relied on the administration of a modified version of the ARM-12, which featured selected, reworded items measuring participants’ perceived working alliance with IBIs. The methodological invariance suggests that the varying degrees of clinician involvement did not significantly affect participants’ interpretation of the construct of working alliance with the IBIs. This also indicates that quantitative comparisons of working alliance with the IBIs across the unguided and guided interventions are possible and meaningful. It is worth noting that working alliance with IBIs may be different from working alliance with only clinicians [41]; thus, additional research is needed to further elucidate the nature of patients’ ratings of working alliance with IBIs and their comparability to working alliance ratings for clinicians in face-to-face psychotherapy.

### Trajectories of Working Alliance

We hypothesized that working alliance would increase over time but found that working alliance followed a quadratic pattern and remained relatively stable in the U-IBI and the G-IBI-Video conditions while displaying significant deterioration in the G-IBI-Text condition. This indicated that working alliance with the IBIs may have been established quickly in the initial weeks. The stable, quadratic pattern of working alliance also corresponds to patterns reported in the literature on face-to-face therapy, suggesting that these patterns may not be unique to IBIs [17].

Nonetheless, the linear decrease in working alliance in the G-IBI-Text condition was surprising. Although deteriorating patterns have been documented in previous studies examining the development of working alliance over the course of face-to-face interventions [42], this trajectory had been rarely detected in working alliance in IBIs. Participants in the G-IBI-Text condition may have established higher expectations for human connection than participants in the U-IBI program; nonetheless, they received less visual and vocal communication with clinicians than participants in the G-IBI-Video condition. The gap between expectation for human connection and the lack of video-based communication may contribute to decreases in feelings connected in the G-IBI-Text condition. Future studies could shed additional light on this finding by examining whether text-based clinician support may interact with changes in treatment expectations, patient role expectation, or other relationship factors to contribute to decreases in working alliance over time.

### Associations Between Working Alliance and Treatment Outcomes

Our results indicated that working alliance mattered in the G-IBI-Video condition. After controlling for baseline levels of symptoms, higher initial levels of working alliance predicted lower depressive symptoms and less functional impairment at 1-month follow-up. In addition, greater increases in working alliance over time predicted lower worry at the end of treatment and at 1-month follow-up. These results are consistent with the extensive literature on face-to-face therapy [2] as well as the literature on IBIs [11], supporting the robust positive relationship between working alliance and positive treatment outcomes. Our novel design allowed us to show that both the initial levels and the trajectories of working alliance contributed to better treatment outcomes separately for different treatment outcomes. In addition, these prospective associations between working alliance and treatment outcomes were detected at the 1-month follow-up, suggesting at least some level of sustainability for these treatment effects.

By contrast, working alliance was not consistently associated with treatment outcomes in the U-IBI or the G-IBI-Text conditions. These findings were unexpected, although not completely inconsistent with previous studies that did not find significant relationships between CBT-based IBIs and working alliance [43-45]. It is possible that the associations between working alliance and treatment outcomes are only present when clinician support is delivered through video-based modalities, the condition most closely aligned with traditional psychotherapy, which includes visual images, nonverbal facial expressions, and varying voice tones. These findings raise an important issue about the function of working alliance in asynchronous IBIs—although the interpretation and ratings of working alliance were similar across IBIs with or without clinician support, working alliance may only help reduce mental health symptoms when clinician support is present through video-based channels (vs text-based channels or no clinician support). Such findings should be replicated in future studies to compare how different communication modalities may influence the associations between working alliance and treatment outcomes.

### Limitations and Future Directions

The study’s findings should be considered in light of the following limitations. First, our sample was composed of individuals with high educational achievement, which may limit the generalizability of these findings. Future research should attempt to replicate these findings with a sample representative of the general clinical population. Second, working alliance in IBIs may be particularly important for individuals with more moderate to severe levels of psychopathology; hence, future studies should examine whether the associations between working alliance and treatment outcomes among IBIs may vary depending on clinical populations. Third, the *MyCompass* program delivered in this study is a 7-week intervention. The brief duration of the interventions may have interfered with participants’ ability to display more complex trajectories of working alliance, which may have been observed in longer interventions. Fourth, this study focused on using CBT-based IBIs to treat subclinical levels of depression, anxiety, and stress; therefore, the findings may not be generalizable to other IBIs using different theoretical frameworks or targeting other symptoms. In addition, this study selected 3 treatment outcomes that were moderately to highly correlated to each other. Future studies should select treatment outcome measures to reduce multicollinearity and examine whether the predictions of working alliance on treatment outcomes may differ depending on the outcome variables.

Future studies should also examine the potential mediators through which the initial level or the change rate of working alliance may affect treatment outcomes. For example, it is possible that an initial high level of working alliance would help the patient to use IBIs more frequently or complete the homework more often, which may result in greater symptom reduction. Finally, the study is limited to assessing working alliance with IBIs; future studies are needed to understand similarities and differences between working alliance with only clinicians and working alliance with IBIs.

### Conclusions

This study examined the conceptual equivalence, trajectories, and outcome associations of working alliance in a randomized controlled trial with 3 conditions, including unguided IBIs as well as guided IBIs with text-based and video-based support. We found conceptual equivalence of working alliance with the IBIs across the 3 conditions. Our results also revealed a quadratic pattern of working alliance over time in the U-IBI and G-IBI-Video conditions, but a deterioration pattern was revealed in the G-IBI-Text condition. Higher initial-level and faster increases of working alliance in the G-IBI-Video condition predicted lower mental health symptoms and functional impairment at the end of treatment and 1-month follow-up compared with the other 2 conditions. Working alliance was also not consistently associated with treatment outcomes in the U-IBI or G-IBI-Text conditions. Our results suggested that despite similar conceptual interpretation and trajectories, the function of working alliance may differ among IBIs with varying degrees and types of clinician support for high-functioning populations with subclinical levels of distress.

## Appendix

Multimedia Appendix 1 MyCompass interface examples.

Multimedia Appendix 2 CONSORT(Consolidated Standards of Reporting Trials) flow chart of participants.

Multimedia Appendix 3 Participants’ demographic information.

## Abbreviations

ARM-12: Agnew Relationship Measure, 12-item version
CBT: cognitive behavioral therapy
CONSORT: Consolidated Standards of Reporting Trials
G-IBI-Text: guided internet-based intervention with text-based clinician support
G-IBI-Video: guided internet-based intervention with video-based clinician support
IBI: internet-based intervention
PHQ-9: Patient Health Questionnaire-9
PSWQ: Penn State Worry Questionnaire
RMSEA: root mean squared error of approximation
SDS: Sheehan Disability Scale
U-IBI: unguided internet-based intervention
