# Supplementary material for: Conceptual Invariance, Trajectories, and Outcome Associations of Working Alliance in Unguided and Guided Internet-Based Psychological Interventions: Secondary Analysis of a Randomized Controlled Trial
Source: JMIR Ment Health. 2022 Jun 21;9(6):e35496. doi: 10.2196/35496 (PMC9257617; doi:10.2196/35496)
Supplement: Multimedia Appendix 1 [file mental_v9i6e35496_app1.docx]

**Multimedia Appendix 1. MyCompass interface examples.**


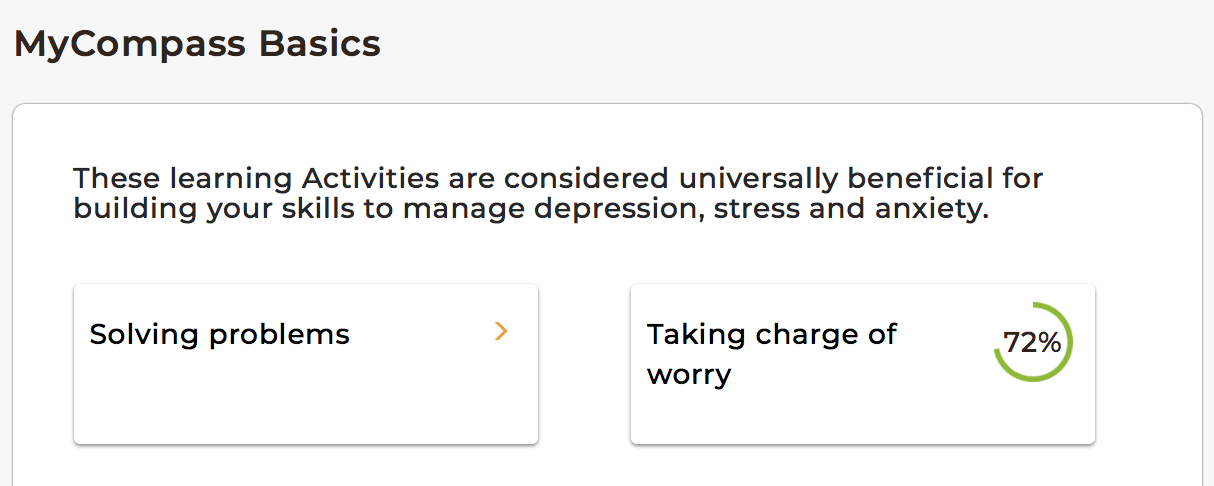


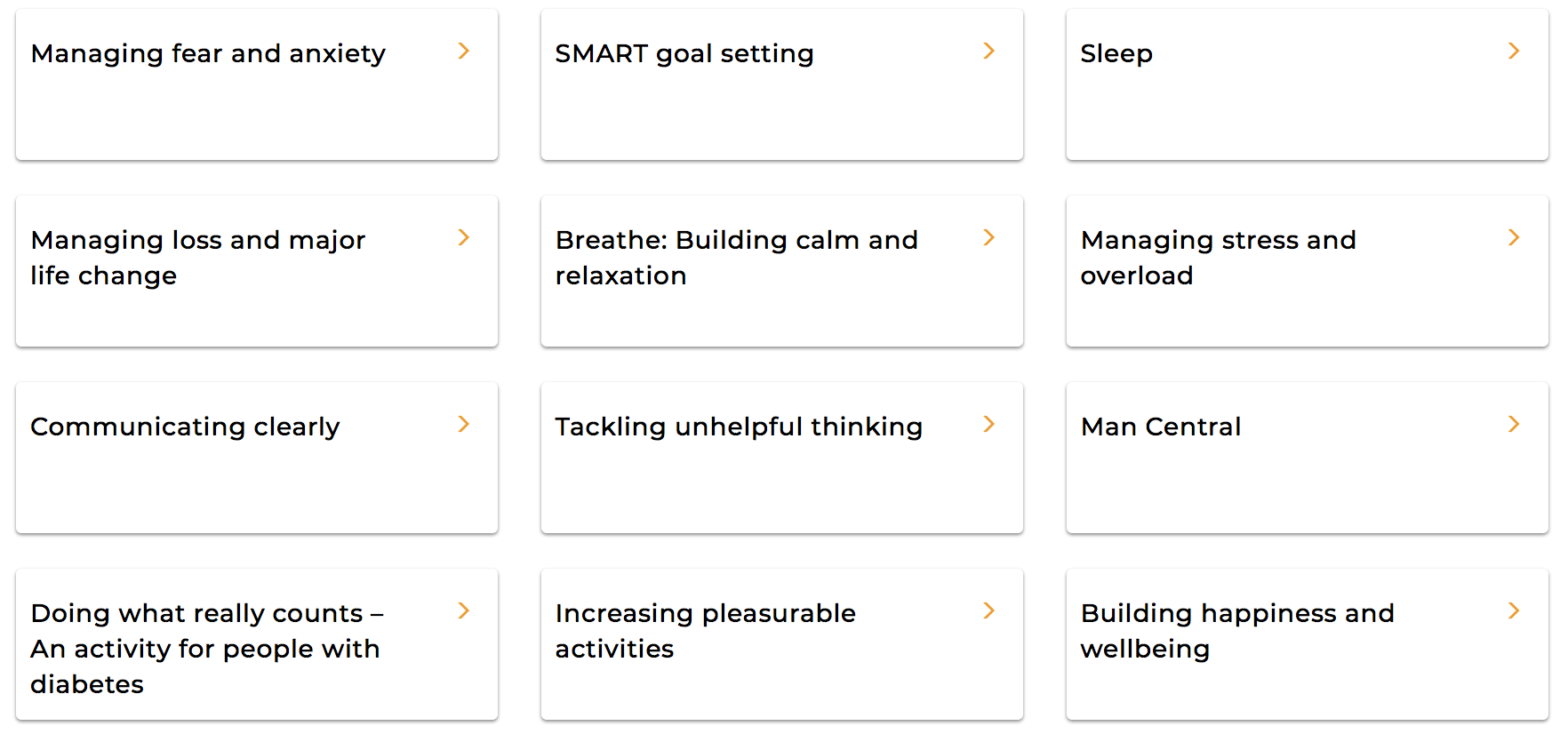


Supplement Figure S1 The MyCompass interface for selecting target concerns


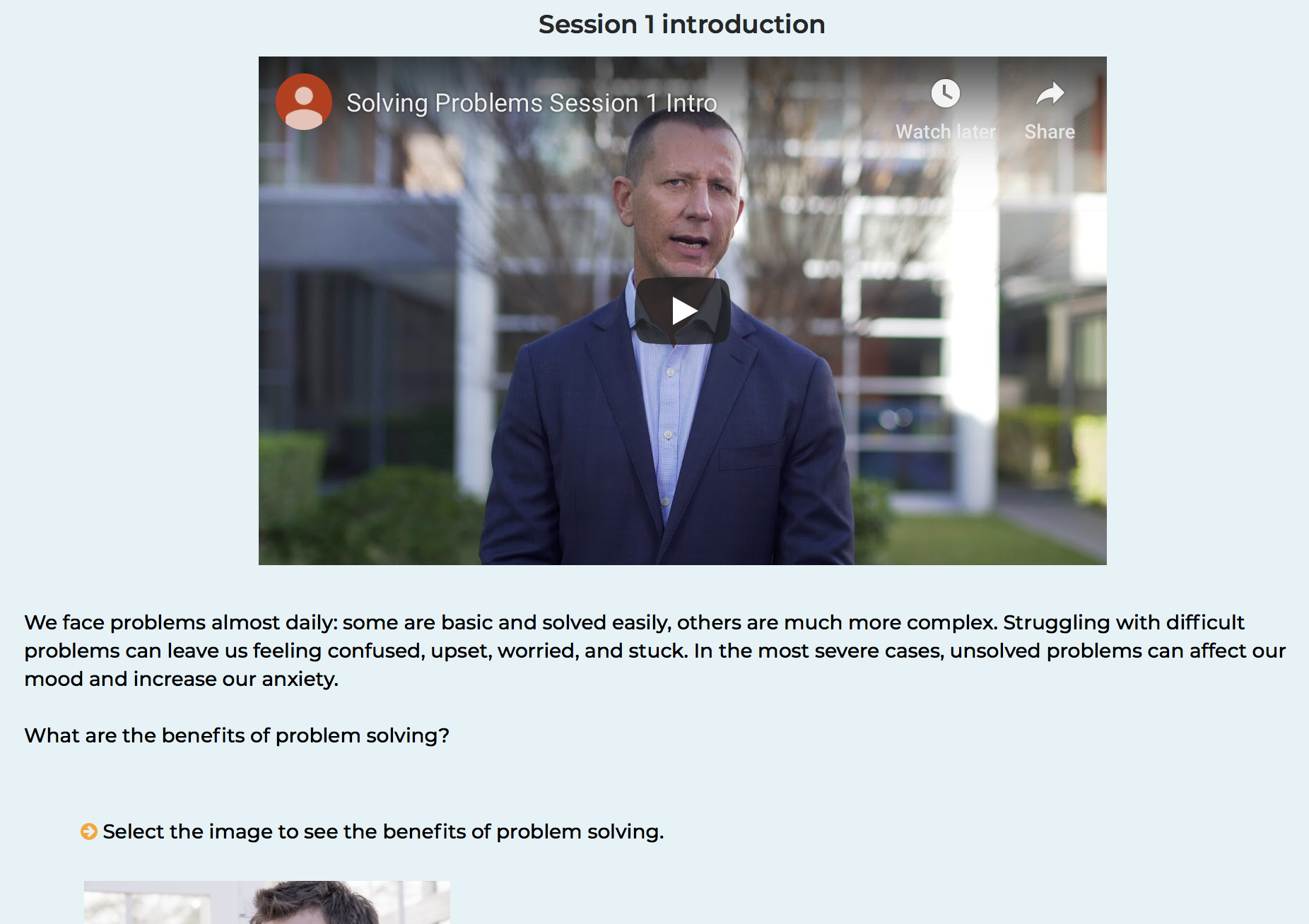


Supplement Figure S2 Examples of the MyCompass modules


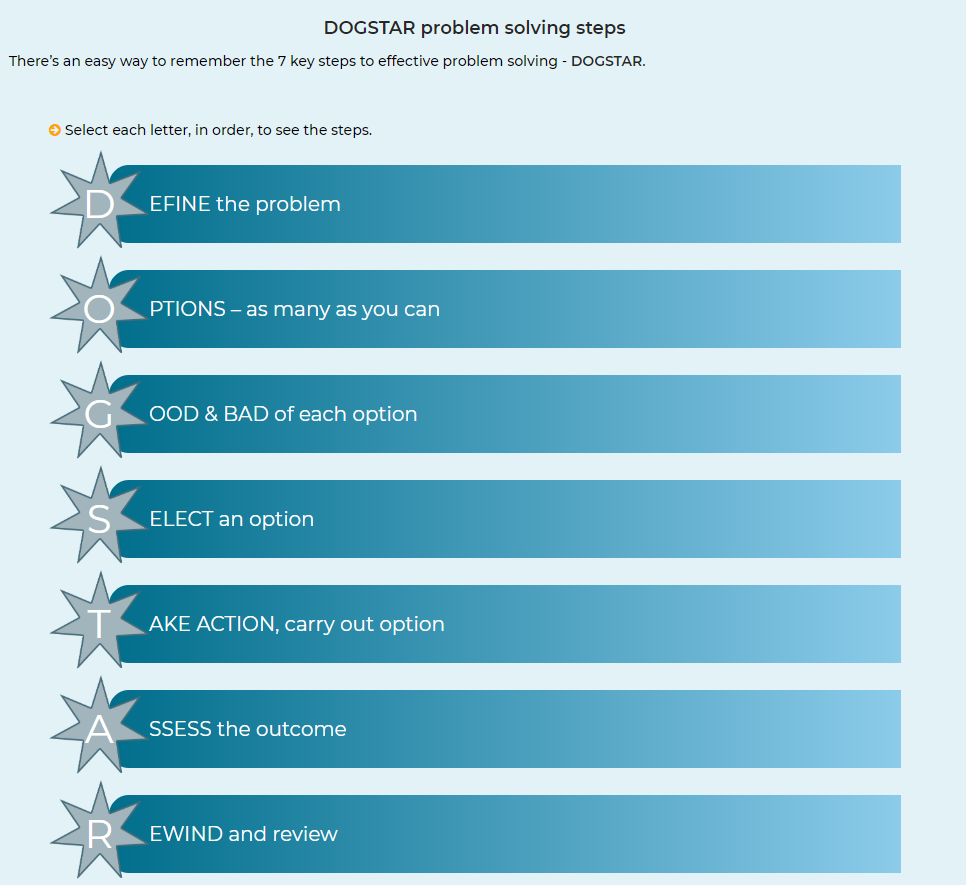


Supplement Figure S3 Examples of the MyCompass modules


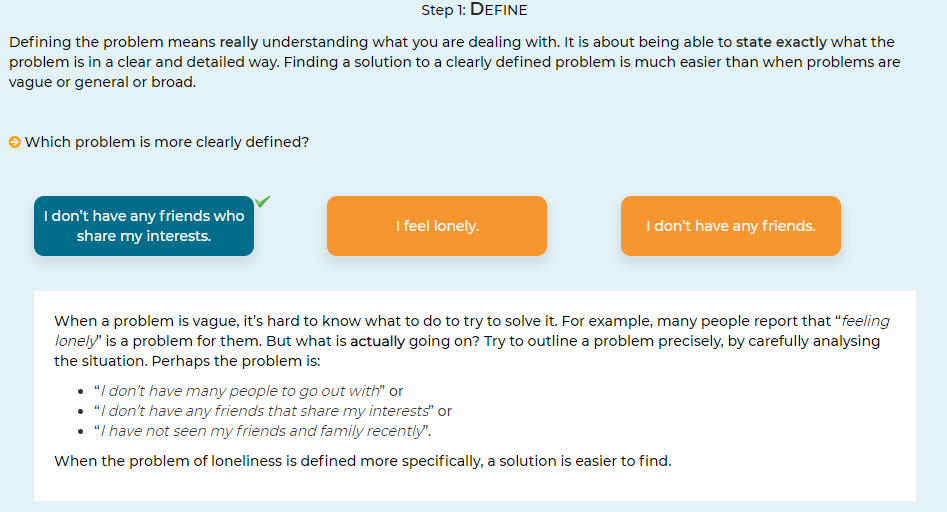


Supplement Figure S4 Examples of the MyCompass modules
