# Supplementary material for: Conceptual Invariance, Trajectories, and Outcome Associations of Working Alliance in Unguided and Guided Internet-Based Psychological Interventions: Secondary Analysis of a Randomized Controlled Trial
Source: JMIR Ment Health. 2022 Jun 21;9(6):e35496. doi: 10.2196/35496 (PMC9257617; doi:10.2196/35496)
Supplement: Multimedia Appendix 2 [file mental_v9i6e35496_app2.docx]

**
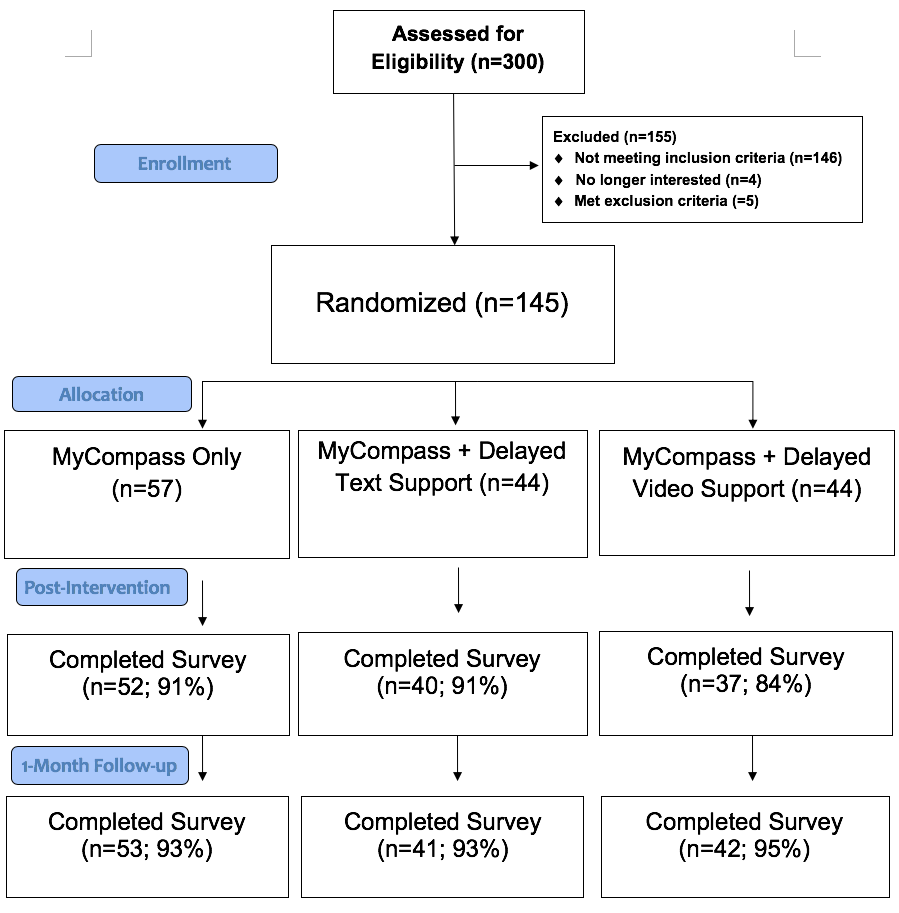
Multimedia Appendix 2. CONSORT(Consolidated Standards of Reporting Trials) flow chart of participants.**
