# Supplementary material for: Conceptual Invariance, Trajectories, and Outcome Associations of Working Alliance in Unguided and Guided Internet-Based Psychological Interventions: Secondary Analysis of a Randomized Controlled Trial
Source: JMIR Ment Health. 2022 Jun 21;9(6):e35496. doi: 10.2196/35496 (PMC9257617; doi:10.2196/35496)
Supplement: Multimedia Appendix 3 [file mental_v9i6e35496_app3.docx]

|  | No. (%) | | | |
| --- | --- | --- | --- | --- |
|  | | U-IBI  n = 57 | G-IBI-TEXT  n = 44 | G-IBI-VIDEO  n = 44 |
| Female Sex | | 41 (72%) | 31 (71%) | 27 (61%) |
| Age (SD) | | 30.91 (7.96) | 30.23 (8.91) | 28.86 (7.86) |
| Sexual Orientation | |  |  |  |
| Heterosexual | | 51 (89.5%) | 35 (79.5%) | 39 (88.6%) |
| Lesbian or Gay | | 1 (1.8%) | 1 (2.3%) | 4 (9.1%) |
| Bisexual | | 2 (3.5%) | 4 (9.1%) | 1 (2.3%) |
| Other | | 3 (4.3%) | 4 (9.1%) | 0 (0%) |
| Race/Ethnicity | |  |  |  |
| White | | 37 (64.9%) | 31 (70.5%) | 31 (70.5%) |
| Asian | | 13 (22.8%) | 9 (20.5%) | 8 (18.2%) |
| Black | | 1 (1.8%) | 2 (4.5%) | 1 (2.3%) |
| Latinx/Hispanic (non-White) | | 4 (7.0%) | 0 (0%) | 7 (15.9%) |
| Other | | 2 (3.5%) | 2 (4.5%) | 2 (4.5%) |
| Multi-racial | | 4 (7.0%) | 0 (0%) | 2 (4.5%) |
| Education | |  |  |  |
| High school | | 2 (3.5%) | 0 (0%) | 0 (0%) |
| Some college | | 1 (1.8%) | 1 (2.3%) | 1 (2.3%) |
| Bachelor’s degree | | 18 (31.6%) | 16 (36.4%) | 20 (45.5%) |
| Graduate degree | | 36 (63.1%) | 26 (59.1%) | 22 (50.1%) |
| Other | | 0 (0%) | 1 (2.3%) | 1 (2.3%) |

**Multimedia Appendix 3. Participants’ demographic information.**
